# Response of Endolithic *Chroococcidiopsis* Strains From the Polyextreme Atacama Desert to Light Radiation

**DOI:** 10.3389/fmicb.2020.614875

**Published:** 2021-01-18

**Authors:** María Cristina Casero, Carmen Ascaso, Antonio Quesada, Hanna Mazur-Marzec, Jacek Wierzchos

**Affiliations:** ^1^Grupo de Ecología y Geomicrobiología del Sustrato Lítico, Departamento de Biogeoquímica y Ecología Microbiana, Museo Nacional de Ciencias Naturales, CSIC, Madrid, Spain; ^2^Departamento de Biología, Universidad Autónoma de Madrid, Madrid, Spain; ^3^Division of Marine Biotechnology, University of Gdańsk, Gdynia, Poland

**Keywords:** *Chroococcidiopsis*, endolithic, Atacama, light, scytonemin

## Abstract

Cyanobacteria exposed to high solar radiation make use of a series of defense mechanisms, including avoidance, antioxidant systems, and the production of photoprotective compounds such as scytonemin. Two cyanobacterial strains of the genus *Chroococcidiopsis* from the Atacama Desert – which has one of the highest solar radiation levels on Earth- were examined to determine their capacity to protect themselves from direct photosynthetically active (PAR) and ultraviolet radiation (UVR): the UAM813 strain, originally isolated from a cryptoendolithic microhabitat within halite (NaCl), and UAM816 strain originally isolated from a chasmoendolithic microhabitat within calcite (CaCO_3_). The oxidative stress induced by exposure to PAR or UVR + PAR was determined to observe their short-term response, as were the long-term scytonemin production, changes in metabolic activity and ultrastructural damage induced. Both strains showed oxidative stress to both types of light radiation. The UAM813 strain showed a lower acclimation capacity than the UAM816 strain, showing an ever-increasing accumulation of reactive oxygen species (ROS) and a smaller accumulation of scytonemin. This would appear to reflect differences in the adaptation strategies followed to meet the demands of their different microhabitats.

## Introduction

Intense solar radiation may trigger alterations in the structure and activity of proteins, DNA and lipids, leading to the inhibition of cell growth and division, pigment bleaching, reduced N_2_ metabolism, and reduced energy production and or photosynthesis (Sinha and Häder, 2008; Rastogi et al., 2014a). UVB radiation (280–320 nm) has the greatest potential for cell damage since it has direct effects on DNA and proteins (Gao and Garcia-Pichel, 2011) while UVA radiation (320–400 nm), produces indirect effects through the production of highly active oxidizing agents such as reactive oxygen species (ROS) (Rastogi and Incharoensakdi, 2014). Even light in the photosynthetically active radiation (PAR) spectrum can have negative effects when intense, e.g., by reducing the rate of photosynthesis and inducing the bleaching of pigments (Han et al., 2003).

Since cyanobacteria originated in the Precambrian era, when the ozone shield was absent, UVR has presumably acted as an evolutionary pressure leading to the development of different protection mechanisms (Rahman et al., 2014) including avoidance, the scavenging of ROS by antioxidant systems, the synthesis of UV-screening compounds, and DNA repair systems for UV-induced DNA damage and protein resynthesis (Rastogi et al., 2014a). Cyanobacteria rely on avoidance as their first line of defense against UVR. Those inhabiting aquatic ecosystems may migrate to deeper parts of the water column where light intensities are lower (Reynolds et al., 1987), terrestrial species, can glide within the structure of a microbial mat to a safer position (Quesada and Vincent, 1997; Büdel, 1999). Another option for terrestrial species is to or colonize endolithic habitats (Wierzchos et al., 2018) as some do in the Atacama Desert. This desert has some of the highest surface radiation levels on Earth, a consequence of its proximity to the equator, high altitude, relatively low ozone column values, prevalently cloudless conditions, and low aerosol loading (McKenzie et al., 2015; Cordero et al., 2016). Indeed, the Atacama Desert reaches UV index values up to 20 (Cordero et al., 2014); the World Health Organization regards a value of 11 to pose an “extreme risk of harm.” So great is the problem that epilithic (rock surface-inhabiting) microbial communities are absent over most of the hyper-arid region of this desert (Cockell et al., 2008). However, just a few millimeters of rock over endolithic microbial communities can drastically reduce the UVR damage that would otherwise occur (Cockell et al., 2003); indeed, just 0.1–2.5% of the total incident solar radiation might reach endolithic habitats (Nienow et al., 1988; Wierzchos et al., 2015).

As a second line of defense, cyanobacteria have developed complex antioxidant systems to cope with UVR-induced oxidative stress (Singh et al., 2013) and the response of several genera from diverse habitats to such stress has been reported (Rastogi et al., 2013; Singh et al., 2014; Baqué et al., 2016; Soule et al., 2016; Han et al., 2019; Muhetaer et al., 2020). Enzymatic antioxidants may include superoxide dismutase (SOD), catalase (CAT), glutathione peroxidase (GPX), and the enzymes involved in the ascorbate-glutathione cycle. SOD protects different cellular proteins against oxidative stress and exists in four different metalloforms: Fe-SOD, Mn-SOD, Cu/Zn-SOD, and Ni-SOD (Priya et al., 2007).

A third line of defense involves the synthesis of UV-absorbing and/or UV-screening compounds (Cockell and Knowland, 1999; Quesada et al., 2001), the most important being mycosporine-like amino acids (MAAs) and scytonemin. MAAs, which have an absorption spectrum of 310–362 nm, are produced by cyanobacteria but also by eukaryotic organisms such as fungi, microalgae and lichens, and accumulated by both invertebrate and vertebrate (Sinha et al., 2007). Scytonemin, the most widespread sunscreen pigment found in cyanobacteria – and which is certainly manufactured by some endolithic forms from the Atacama Desert (Vítek et al., 2014, 2016, 2017; Wierzchos et al., 2015)- is exclusively produced by these organisms (Rastogi and Incharoensakdi, 2014; Rastogi et al., 2015). A yellow-brown lipid-soluble dimeric compound composed of indolic and phenolic subunits (Proteau et al., 1993), it occurs in oxidized (MW 544 Da) and reduced (MW 546 Da) forms and has an absorption spectrum of 250–450 nm, i.e., covering the wavelengths of UVA and UVB. Its *in vivo* absorption maximum is at 370 nm, while its maximum in purified form is at 386 nm. Located in the exopolysaccharide sheath (EPS), it is highly stable under different abiotic stress conditions and can reduce the UVA radiation penetrating the cell by about 90% (Garcia-Pichel and Castenholz, 1991). Its high stability allows it to persist over very long periods in terrestrial crusts and dried mats (Potts, 1994). It performs its function without any further metabolic investment.

*Chroococcidiopsis* species are extremotolerant organisms, occurring in a variety of terrestrial habitats (Bahl et al., 2011) that avoid high light intensities and UVR by living in the soil, caves, and endolithic habitats (Wierzchos et al., 2018). The UVR tolerance of several strains of *Chroococcidiopsis* has been characterized by exposing them to similar conditions as those occurring on Mars (Cockell et al., 2005; Baqué et al., 2016; Billi et al., 2019). Their production of scytonemin has also been investigated (Dillon and Castenholz, 1999; Dillon et al., 2002; Fleming and Castenholz, 2007).

The aim of the present work was to examine the effects of, and responses to, PAR and UVR + PAR of (1) the *Chroococcidiopsis* strain UAM813 originally isolated from a cryptoendolithic microhabitat within beneath 5–7 mm layer of translucent halite from the Atacama Desert – previously characterized (Wierzchos et al., 2006, 2012; de los Ríos et al., 2010; Robinson et al., 2015) and reported to harbor scytonemin produced by cyanobacteria (Vítek et al., 2014) – and (2) *Chroococcidiopsis* strain UAM816, isolated from the chasmoendolithic habitat within calcite (DiRuggiero et al., 2013) – a microhabitat more exposed to direct solar radiation due to direct contact of cracks and fissures with the rock surface – from the same desert. The results obtained would appear to reflect differences in the adaptation strategies followed to meet the demands of these strains’ different microhabitats.

## Materials and Methods

### Culture Organisms and Conditions

The strains of cyanobacteria used in the present work were *Chroococcidiopsis* UAM813, originally isolated from the cryptoendolithic microhabitat within halite from the Yungay area of the Atacama Desert (24°05′09″ S, 069°55′17″ W) (see Supplementary Figures 1A–C) and *Chroococcidiopsis* UAM816, originally isolated from the chasmoendolithic microhabitat within calcite from the Valle de la Luna in the same desert (22°54′39″ S, 06814′49″ W) (see Supplementary Figures 1D–F). Both strains are maintained at the Universidad Autónoma de Madrid (Madrid, Spain). Both *Chroococcidiopsis* UAM813 and UAM816 (Supplementary Figure 2) were grown in batch culture in BG11 medium (Rippka et al., 1979) at 28°C under 12 W m^–2^ PAR (∼60 μmol photons m^–2^ s^–1^) generated by cool white fluorescent lamps.

Cultures were gently homogenized by orbital shaking and 3 mL aliquots filtered through Cyclopore Track-Etch Membranes (Whatman) (25 mm diameter, pore size 0.2 μm pore size), leaving a thin layer of cells behind (Supplementary Figure 3). These filters were then transferred to 1% agar plates containing BG11 medium and the cells allowed to adjust to the new substrate, for 48 h at 25°C [naturally, these conditions are not the same as those encountered in cryptoendolithic (halite) or chasmoendolithic (calcite) microenvironments].

The strains were exposed to different light conditions, i.e., 40 W m^–2^ PAR, or the same plus 2 W m^–2^ UVA (UVR + PAR) (F20T10/BLB lamp (315–400 nm), for the times described below (Supplementary Figure 4). Culture surface measurements of PAR (400–700 nm) and UVR (215–400 nm) intensity were checked using an ULM-500 universal light meter (Heinz Walz GmbH, Effeltrich, Germany) and Apogee UV Radiation MU-200 meter (Apogee Instruments, Logan, UT, United States), respectively.

All described experiments were performed in triplicates following the Fleming and Castenholz (2007) indications.

### Short-Term Effects: *In vivo* Detection of Oxidative Stress

To examine the short-term effects of irradiation on the strains, the production of ROS was examined after 0, 24, 48, and 72 h of exposure by staining with 2′,7′-Dichlorodihydrofluorescein diacetate (DCFH-DA) (Sigma-Aldrich – Merck KGaA, Darmstadt, Germany) dissolved in ethanol. The irradiated cells were resuspended in 1 mL of 0.1 M phosphate buffer (PBS) and DCFH-DA added to a final concentration of 5 μM. The samples were then incubated in a shaker at room temperature in the dark for 1 h. DCFH is non-fluorescent but it becomes highly fluorescent DCF when oxidized by intracellular ROS or peroxides; it has an excitation wavelength of 485 nm and an emission band of 500–600 nm. After 1 h of incubation, the fluorescence of the samples was measured using a spectrofluorophotometer at the latter excitation wavelength and emission band settings. The intensity of the fluorescence was corrected against blank controls without cells and normalized to dry weight (DW) using a XP6 microbalance (Mettler Toledo, Columbus, OH, United States).

In addition, CellROX Green reagent (Invitrogen, Waltham, MA, United States) was used to detect the location of ROS by fluorescence microscopy following the method of Cornejo-Corona et al. (2016) optimized for the present conditions. Briefly, cells irradiated on BG11 medium agar plates as above were resuspended in 100 μL of 0.1 M PBS. 2 μL of 5 mM CellROX Green was then added and the samples incubated at room temperature with shaking at 120 rpm for 30 min in the dark. The cells were then washed twice for 5 min at room temperature with 1× PBS, 0.1% Triton X-100. At least one hundred cells (identified by bright field microscopy) were examined for each experimental timepoint. The fluorescence of the detected cells was then observed using a Zeiss Axio Imager M2 fluorescence microscope (Carl Zeiss, Jena, Germany) and an Apochrome x60, *n* = 1.4 Zeiss oil-immersion objective. Images were captured using a Multichannel Image Acquisition system and employing the eGFP filter set (Zeiss Filter Set 38; Ex/Em: 450–490/500–550 nm) for CellROX green fluorescence and weak EPS autofluorescence, and Rhodamine filter set (Zeiss Filter Set 20; Ex/Em: 540–552/567–647 nm) for red chlorophyll *a* and the phycobiliproteins autofluorescence signal.

Although cultures were not axenic, heterotrophic biomass never exceeded 1–2% of the total biomass based on cell counts according to Schallenberg et al. (1989).

### Long-Term Effects: Production of Scytonemin

*Chroococcidiopsis* UAM813 and UAM816 cells were exposed to PAR or UVR + PAR for 0, 3, 6, 9, 12, and 15 days to examine the production of scytonemin. After exposure, the cells were scraped off the filters, and suspended in 1:1 (v/v) methanol: ethyl acetate (M-EA), gently homogenized by pumping them multiple times with a 1000 μL Pipetman (Gilson, Middleton, WI, United States) and incubated overnight at 4°C in darkness to allow for the extraction of scytonemin (Rastogi and Incharoensakdi, 2014). The samples were then centrifuged (10,000 × *g* for 5 min) and the supernatant filtered through 0.2 μm pore-sized sterilized syringe-driven filter (Symta, Madrid, Spain). The M-EA extract was then subjected to high-performance liquid chromatography (HPLC) using an Agilent Technologies 1200 Series, Photodiode Array (PDA) (Agilent, Santa Clara, CA, United States) system.

For these HPLC analyses, 20 μL of M-EA extract were injected into a Phenomenex Peptide HPLC column (100 Å, 3.6 μ × 4.60 mm; XB C18) (Phenomenex, Torrance, CA, United States). The mobile phase was composed of 5% acetonitrile in MilliQ water + 0.1% formic acid (solvent A) plus 100% acetonitrile + 0.1% formic acid (solvent B). A 30 min elution gradient program was set with 0–15 min linear increase from 15 to 80% solvent B, followed by 15–30 min at 100% solvent B. The elution flow rate was 0.5 mL min^–1^. The detection wavelength was 384 nm. The PDA scan wavelength ranged from 200 to 700 nm. Oxidized and reduced scytonemin were distinguished by their characteristic absorption maxima.

In addition, the scytonemin content in the M-EA extracts was determined by measuring the latter’s absorbance at 384 nm (scytonemin maximum). Pooled carotenoids were also determined at 490 nm, and that of chlorophyll *a* at 663 nm. These absorbance values were corrected for residual scatter by subtracting the absorbance at 750 nm. All absorbance measurements were made on a Flame Spectrometer (Ocean Optics, Orlando, FL, United States).

All scytonemin measurements were normalized to DW using a XP6 microbalance as above. Once again, although cultures were not axenic, heterotrophic biomass never exceeded 1–2% of the total biomass based on cell counts according to Schallenberg et al. (1989).

Finally, light microscopy observations of these cells were also made to follow the production of scytonemin via their change in color over time (from blue-green to yellow-brown as more scytonemin accumulates). Observations were made in differential interference contrast (DIC) mode using an Axio Imager M2 microscope (Carl Zeiss, Germany) equipped with Apochrome x64, *n* = 1.4 oil immersion objective.

### Long-Term Effects of Light Exposure on the Metabolic Activity Experiment and Ultrastructure of the Strains

The metabolic activity of *Chroococcidiopsis* cells was determined at 0, 3, 6, 9, 12, and 15 days of PAR or UVR + PAR exposure as above, using the cell-permeable 5-Cyano-2,3-Ditolyl Tetrazolium Chloride (CTC) redox dye. In metabolically active cells, this dye is reduced from its soluble colorless form into insoluble fluorescent formazan (CTF), which accumulates intracellularly as opaque, dark-red crystals which can be detected under bright field illumination, or as yellow-orange fluorescent spots (excitation and emission maxima at 488 and 630 nm) when using fluorescence microscopy. CTC staining was performed according to Tashyreva et al. (2013), increasing the incubation time from 2 to 5 h. Microscopy was performed using a fluorescence microscope (Zeiss Axio Imager M2, Carl Zeiss, Jena, Germany) equipped with Apochrome oil immersion objective x64, *n* = 1.4 and HE Rhodamine filter set (Ex/Em: 426–446/545– 645 nm).

### Transmission Electron Microscopy (TEM)

After light treatment as above, UAM813 and UAM816 cells were resuspended in 3% glutaraldehyde in 0.1 M cacodylate buffer and incubated at 4°C for 3 h. The cells were then washed three times in cacodylate buffer, postfixed in 1% osmium tetroxide for 5 h before being dehydrated in a graded ethanol series and embedded in LR White resin (de los Ríos and Ascaso, 2002). Ultrathin sections were stained with lead citrate and observed with a JEOL JEM-2100 electron microscope (Tokyo, Japan) equipped with a Gatan Orius CCD camera (Pleasanton, CA, United States) at 200 kV.

### Statistical Analysis

All results are presented as means of the three replicates. Results were examined using one-way analysis of variance (ANOVA), Student *t*-test and *post hoc* Tukey test as required. All calculations were made using SPSS Statisticv.26.0 software for Windows (IBM Corp., Armonk, NY, United States).

## Results

### Short-Term Effects of Exposure to Irradiation

#### Intracellular ROS

**FIGURE 1:**
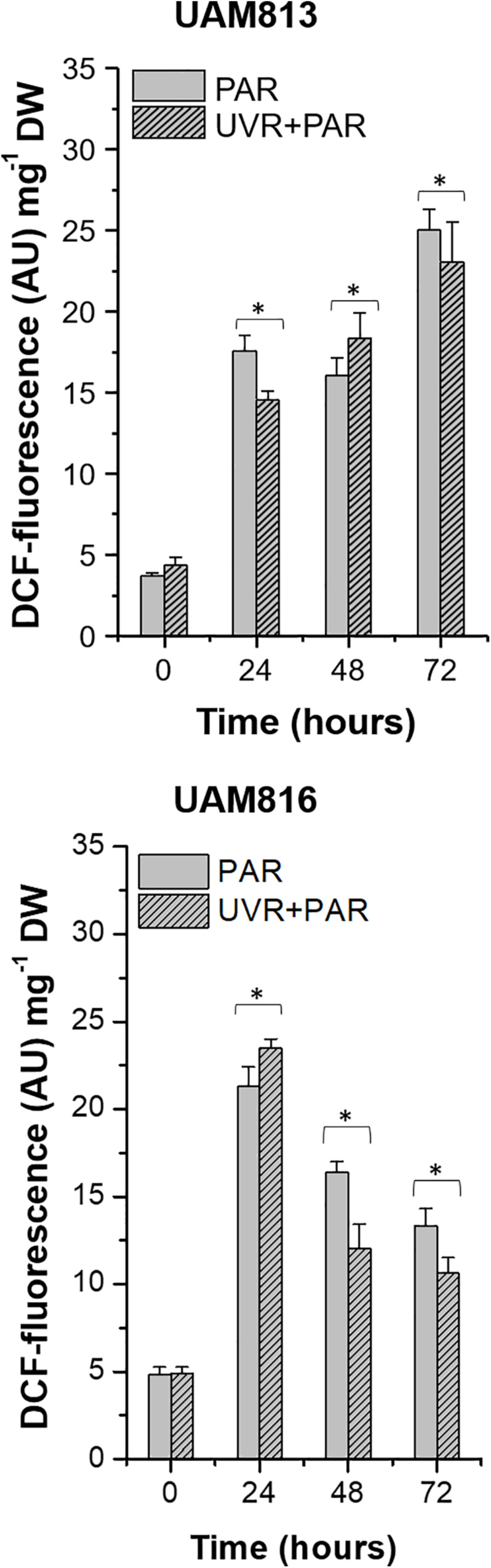
DCF fluorescence in UAM813 **(upper graph)** and UAM816 **(lower graph)** cells after irradiation with PAR (plain bars) or UVR + PAR (lined bars) for 72 h normalized to DW. Error bars indicate standard deviation. The asterisks indicate significant differences between light conditions at each timepoint (*t*-test *p* < 0.05).

In the UAM813 strain, ROS accumulation, represented by DCF fluorescence, increased at 24 h of exposure under both PAR and UVR + PAR, reaching a maximum at 72 h (Figure 1). The light treatments had a significant effect on ROS accumulation at all timepoints (*t*-test *p* < 0.05). Specifically, PAR was associated with a greater accumulation of ROS at 24 and 72 h of exposure than was UVR + PAR, while UVR + PAR conditions induced significantly greater ROS accumulation at 48 h of exposure (Figure 1).

In the UAM816 cells, oxidative stress increased at 24 h of exposure under both treatments, with maximum DCF fluorescence values recorded at this time. A significant reduction in DCF fluorescence was observed at 48 and 72 h. At 24 h, the accumulation of ROS under PAR was smaller than that seen under UVR + PAR. In contrast, at 48 and 72 h of exposure, greater ROS was detected under PAR (*t*-test *p* < 0.05; Figure 1).

**FIGURE 2:**
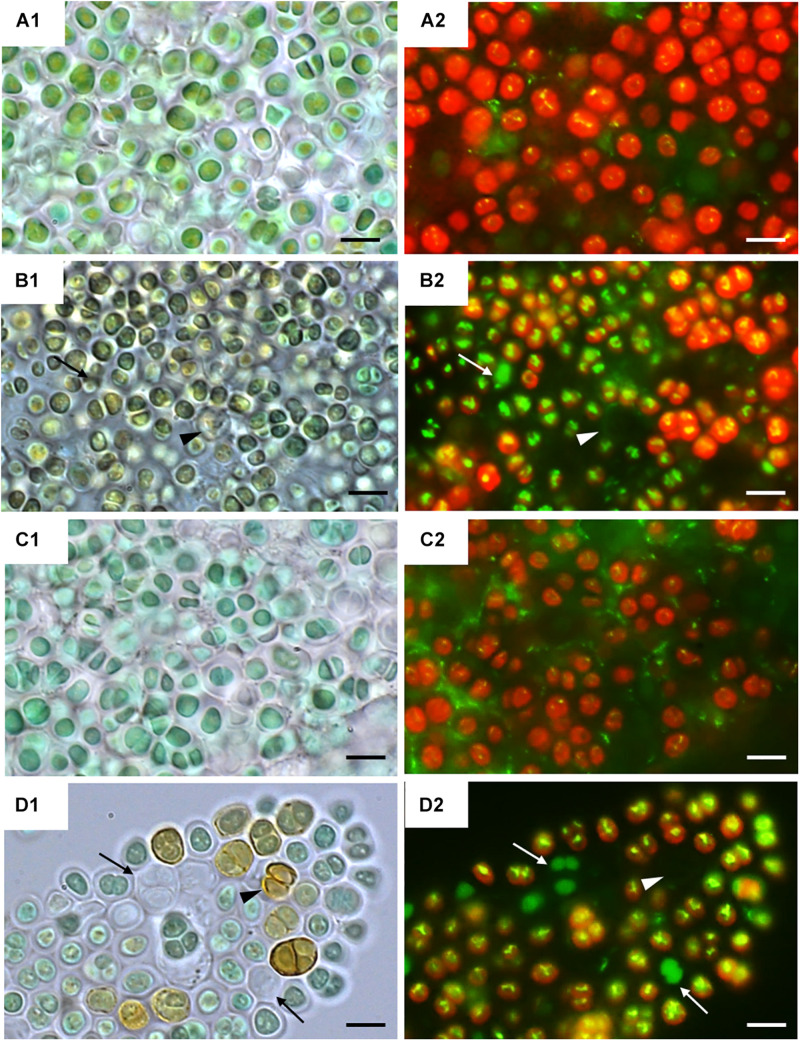
CellROX staining for intracellular detection of ROS in UAM813 (Series A,B) and UAM816 (Series C,D) cells. Bright field **(A1,C1)** and fluorescence microscopy **(A2, C2)** images of Series A,C correspond to exposure time *t* = 0, and in Series B,D after 24 h of exposure to UVR + PAR. The red signal corresponds to cyanobacterial chlorophyll a and phycobiliproteins autofluorescence, which was stronger in PAR only exposed cells **(A2,C2)** than in UVR + PAR exposed cells **(B2,D2)**. Bright yellow/green dots in fluorescence images are CellROX fluorescence staining (oxidative stress indicator); thus, oxidative stress was greater in UVR + PAR exposed cells **(B2,D2)** than in PAR only exposed cells **(A2,C2)**. In images of UVR + PAR-treated cells, the arrows point to cells revealing apparent structural integrity **(B1,D1)** via the green autofluorescence signal, and no autofluorescence either CellROX fluorescence signal **(B2,D2)** and brown color **(B1,D1)** suggesting an increase in scytonemin content. Scale bars = 8 μm.

Figure 2 shows bright field microscopy images (CellROX Green staining) of the strains at 0 h (Figures 2A1,C1) and 24 h of exposure to UVR + PAR (Figures 2B1,D1). The UAM813 and UAM816 cells were light green and blue-green, respectively at 0 h, turning to brownish green and yellow-brown, respectively after 24 h of exposure to UVR + PAR.

Fluorescence microscopy images on Figure 2 show strain UAM813 (Figure 2A2) and UAM816 (Figure 2C2) exhibited intense red autofluorescence of chlorophyll *a* and phycobiliproteins at 0 h. The EPS signal for green autofluorescence was weak at 0 h. After 24 h of exposure to UVR + PAR, the oxidation of CellROX fluorochrome by ROS and its binding to DNA lead to the formation of bright green-yellow fluorescence spots within both the UAM813 (Figure 2B2) and UAM816 (Figure 2D2) cells.

Many of the cells of both strains lost their red autofluorescence signal after irradiation for 24 h with UVR + PAR and emitted only a green autofluorescence signal (white arrows in Figures 2B2,D2). Some cells gave no fluorescence signal at all (arrowheads on Figures 2B2,D2). These were actually just the remaining brown EPS husks of dead cells (black arrowheads on Figures 2B1,D1).

### Long-Term Effects of Exposure to Irradiation

#### Scytonemin Production

No significant difference was seen between values returned by the two scytonemin quantification methods used.

**FIGURE 3:**
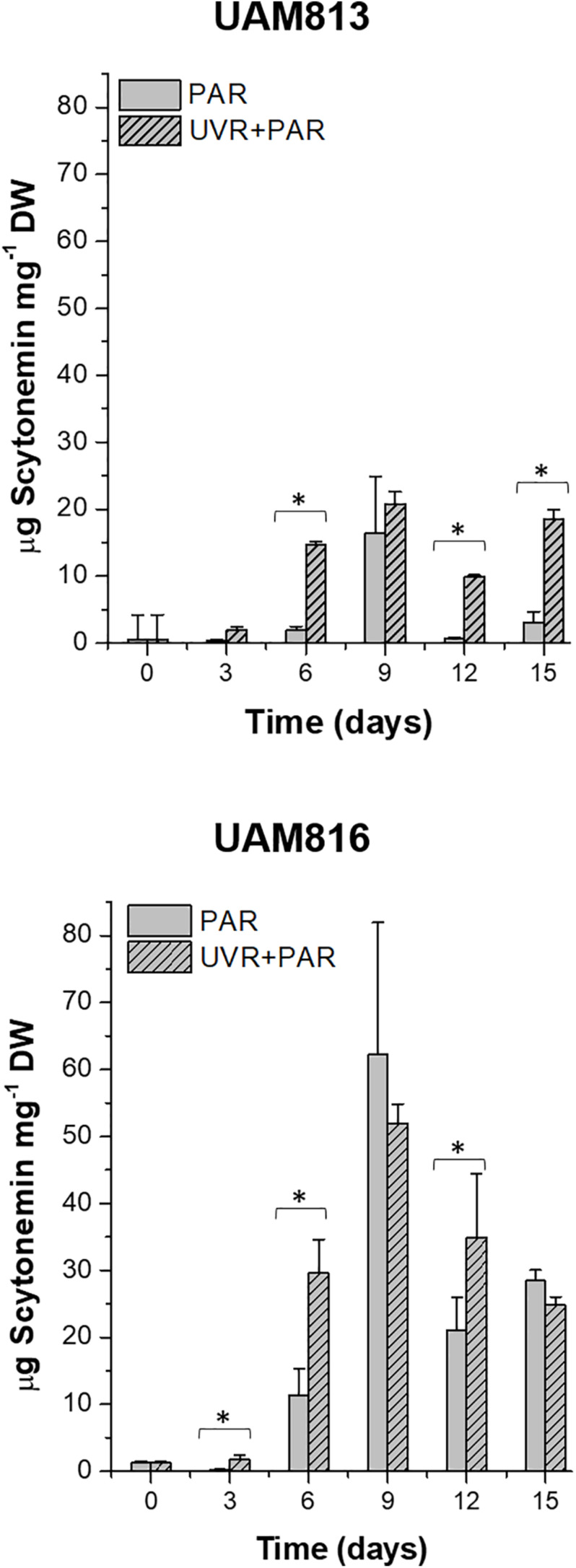
Total scytonemin content, as quantified by HPLC, in UAM813 **(upper graph)** and UAM816 **(lower graph)** cells after irradiation with PAR (plain bars) or UVR + PAR (lined bars) for 15 days normalized to DW. Error bars indicate standard deviation. The asterisks indicate significant differences between light conditions at each timepoint (*t*-test *p* < 0.05).

The scytonemin content in UAM813 strain (Figure 3) increased over the first 9 days of exposure to PAR and UVR + PAR. Under PAR only, a maximum scytonemin content of 16.4 ± 8.4 μg mg^–1^ DW was reached at this timepoint; under UVR + PAR, a maximum scytonemin content of 20.8 ± 1.8 μg mg^–1^ DW was reached. Significant differences in scytonemin content due to the experimental light conditions were observed after only 6 days of exposure (*t*-test *p* < 0.05) when the scytonemin content of the cells under UVR + PAR reached 14.6 ± 0.6 μg mg^–1^ DW but was just 1.9 ± 0.6 μg mg^–1^ DW under PAR only. Significant treatment-associated differences in scytonemin were also observed during the last 3 days of experiment. After 12 days under PAR only, the scytonemin content had reduced to pre-exposure levels. However, under UVR + PAR, no significant differences in scytonemin content were seen between the day 12 and day 9 values (*t*-test *p* < 0.05).

The scytonemin content in the UAM816 strain (Figure 3) reached a maximum after 9 days under PAR and UVR + PAR, with no significant differences between them (62.3 ± 16.6 μg mg^–1^ and 52 ± 2.9 μg mg^–1^ DW, respectively; *t*-test *p* < 0.05). Under both types of light, the scytonemin content fell during the last 6 days of exposure. Significant differences in scytonemin content were seen under the different experimental conditions at 3, 6, and 12 days of exposure (*t*-test *p* < 0.05). The greatest difference between the treatments was seen at 6 days of exposure, with the scytonemin content reaching 29.6 ± 5.1 μg mg^–1^ DW under UVR + PAR and 11.4 ± 3.9 μg mg^–1^ DW under PAR.

The HPLC analysis of the scytonemin showed two prominent peaks in chromatograms of both *Chroococcidiopsis* strains (Supplementary Figures 5A,B) at retention time 16.57 min (a) and the other at 17.89 min (b). The UV absorption maximum at 385 nm identified both reduced (a) and oxidized scytonemin (b) (see Rastogi and Incharoensakdi, 2014). However, the proportion of each type of accumulated cannot be known given the presence of O_2_ in the atmosphere during the extraction procedure; some of the reduced scytonemin may have become oxidized.

#### Long-Term Effects of PAR and UVR + PAR in Metabolic Activity Micromorphology and Ultrastructure

The metabolic activity of the *Chroococcidiopsis* cells was evaluated after 15 days of exposure to two different light conditions, PAR and UVR + PAR.

**FIGURE 4:**
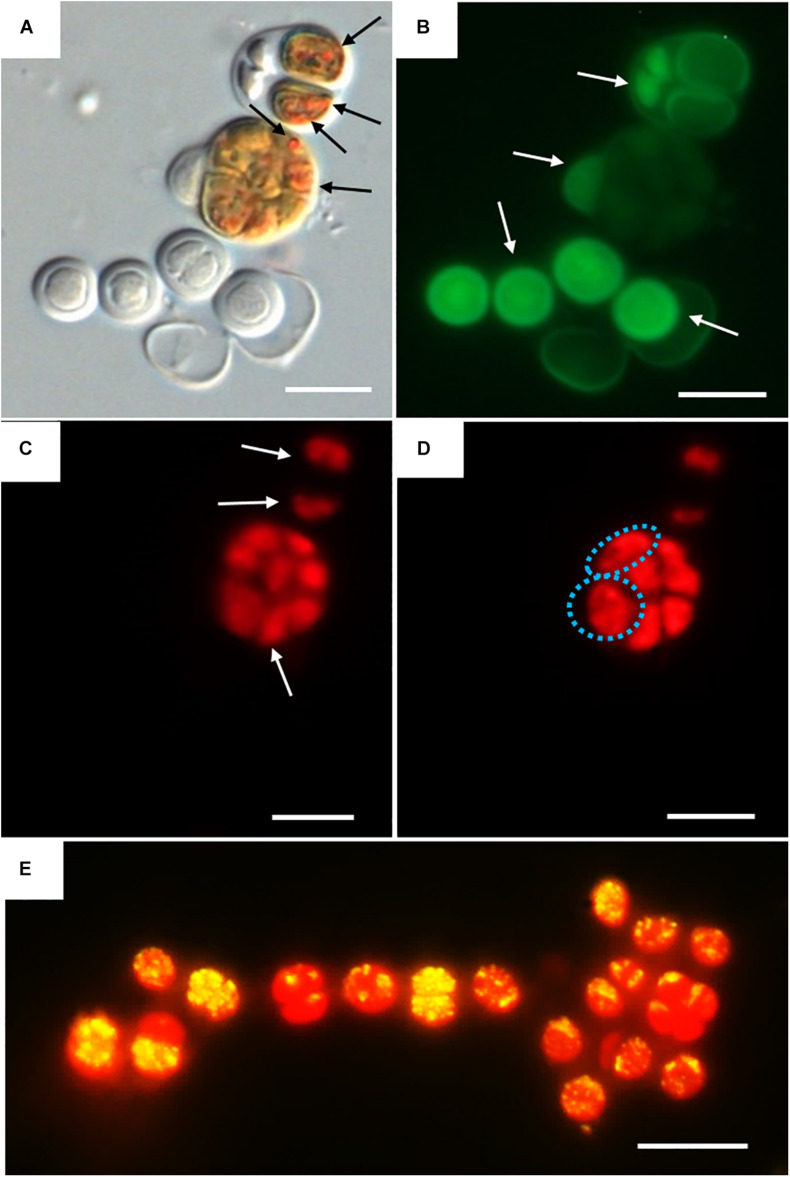
DIC and fluorescence microscopy showing examples of metabolic activity in UAM816 cells after 6 days of irradiation with UVR + PAR. **(A)** DIC microscopy image with orange CTF crystals in metabolically active cells (black arrows). **(B)** eGFP filter set fluorescence image revealing unviable cells (GF+) (white arrows). **(C)** HE rhodamine filter set fluorescence image revealing cells with phycobiliproteins and chlorophyll *a* autofluorescence (white arrows) (PCHL+). **(D)** HE rhodamine filter set fluorescence image of cells with phycobiliproteins and chlorophyll *a* autofluorescence and weak CTF fluorescence (granulose red fluorescence) (blue dotted cells) (PCHL+/CTC+). **(E)** HE rhodamine filter set fluorescence image of UAM816 cells after 3 days of under UVR + PAR. Cells with phycobiliproteins and chlorophyll *a* red autofluorescence plus a yellow signal indicating strong metabolic activity (PCHL+/CTC+). Scale bars = 10 μm.

Three categories were established to describe the vital status of the cells following CTC staining depending on their fluorescence emission. Those with green autofluorescence (GF+) were defined as unviable according to Roldán et al. (2014). Those exhibiting only red chlorophyll *a* and phycobiliproteins autofluorescence (PCHL+) were defined as damaged (not metabolically active); while cells presenting both chlorophyll *a* and phycobiliproteins red autofluorescence and bright orange spots (CTF crystals) (PCHL+/CTC+) were defined as metabolically active (Figure 4).

**FIGURE 5:**
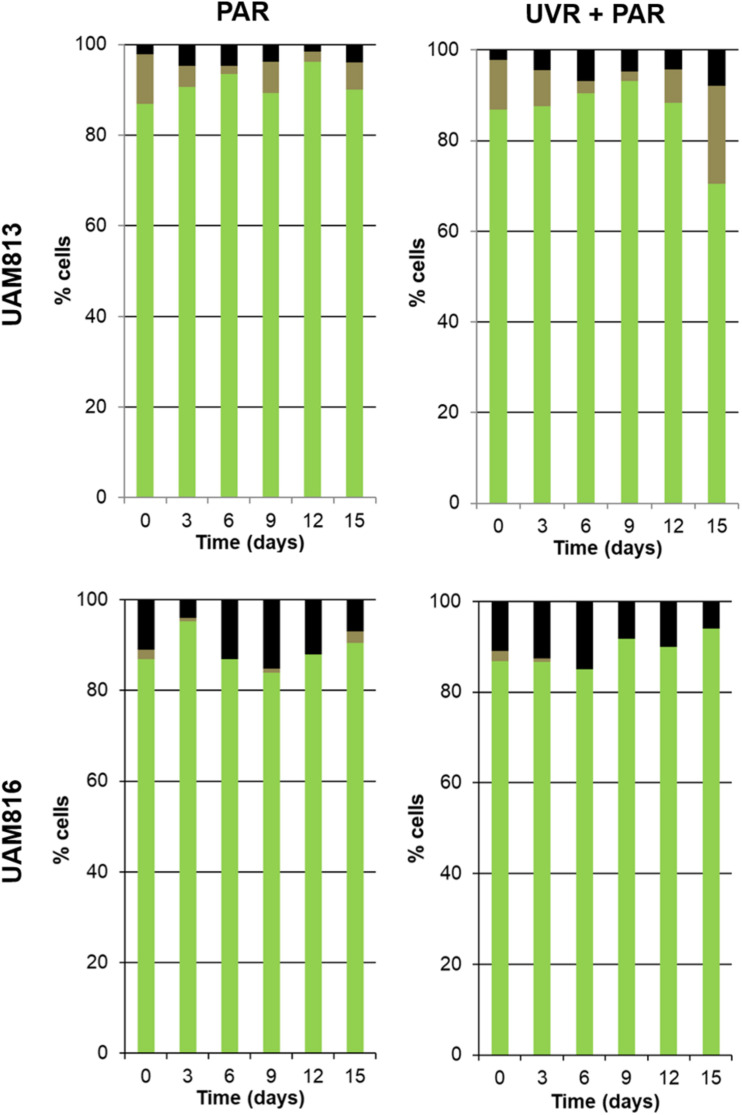
Metabolic activity of UAM813 and UAM816 cells after irradiation with PAR **(left graphs)** or UVR + PAR **(right graphs)** for 15 days. Green: (PCHL+/CTC+) active cells. Brown: (PCHL+) damaged cells. Black: (GF+) unviable cells.

For the UAM813 strain (Figure 5), 86.9–96.2% of cells were active during the first 12 days of exposure to PAR. By day 15, however, 90.1 ± 2.2% were active. Under both PAR and UVR + PAR, after the maximum abundance of active cells was reached at 9 days of exposure (93.2 ± 1.8%), at 12 and 15 days, however, this figure had fallen to 70.5 ± 2.3%, i.e., below the recorded starting value. Under both PAR and UVR + PAR, the maximum percentage of damaged cells was recorded at 15 days (5.9 ± 0.9% under PAR and 21.6% under UVR + PAR), while the relative abundance of unviable cells reached its maximum after 6 days under PAR (4.7 ± 0.4%) and 15 days under UVR + PAR (7.9 ± 0.4%). The light treatments had a significant effect on the relative abundance of active cells all timepoints (*t*-test *p* < 0.05).

Under PAR, the metabolic activity of the UAM816 cells (Figure 5) reached a maximum after 3 days (95.2 ± 1.7%; 86–90% at following experimental times). Under UVR + PAR it was reached at 15 days (94 ± 2.1%) following a progressive increase over time. No damaged cells were seen, whereas under PAR 2.5 ± 0.4% of cells were damaged on day 15. Unviable cells, however, were detected at all times under both light conditions. After exposure to PAR for 9 days, a maximum 15.1 ± 0.7% unviable cells were seen, reducing to 7 ± 0.4% at 15 days. Similar proportions were recorded under UVR + PAR, with a maximum of 15 ± 0.3% unviable cells at 9 days and 6 ± 0.5% at 15 days. The light treatments had a significant effect on the relative abundance of active cells at 3, 9, 12, and 15 days (*t*-test *p* < 0.05).

**FIGURE 6:**
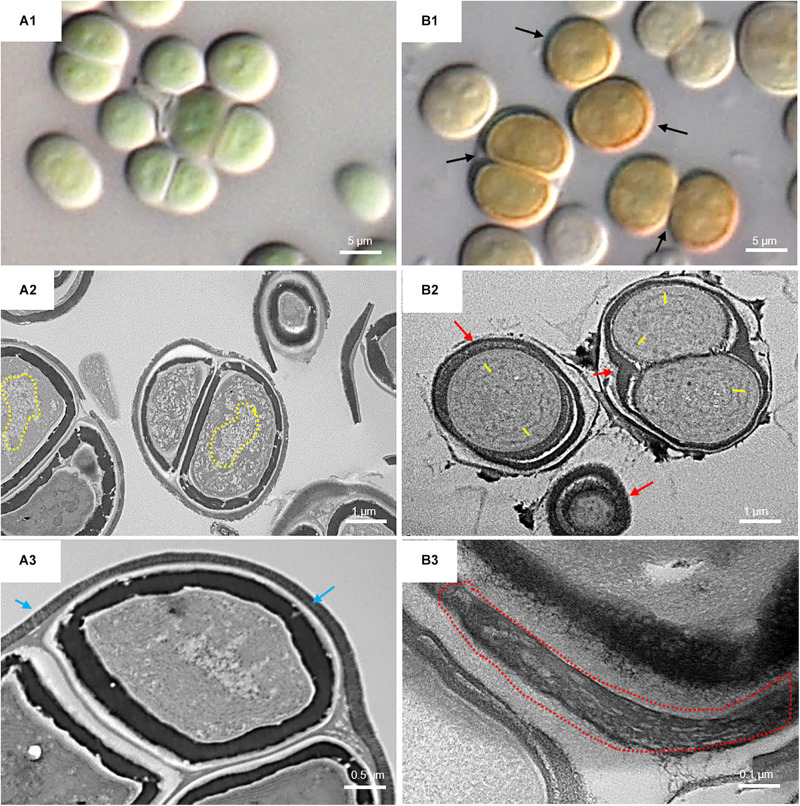
DIC microscopy and TEM images of UAM813. Series A: Cells and aggregates at the beginning of the experiment. **(A1)** Green UAM813 cells. **(A2)** TEM image of cells with a visible nucleoid area (yellow dotted line). **(A3)** TEM image with a cell aggregate showing a thin, outermost fibrous layer (blue arrows). Series B: Cells and aggregates with maximum scytonemin content after 9 days of irradiation with UVR + PAR. **(B1)** Brownish UAM813 cells with higher scytonemin content at the edge. **(B2)** TEM image of cells showing separated thylakoids (yellow lines) and an electron dense outermost fibrous layer (red arrows). **(B3)** TEM image of the outer part of the cells from the same aggregate revealing a highly fibrous outermost layer (red dotted line).

**FIGURE 7:**
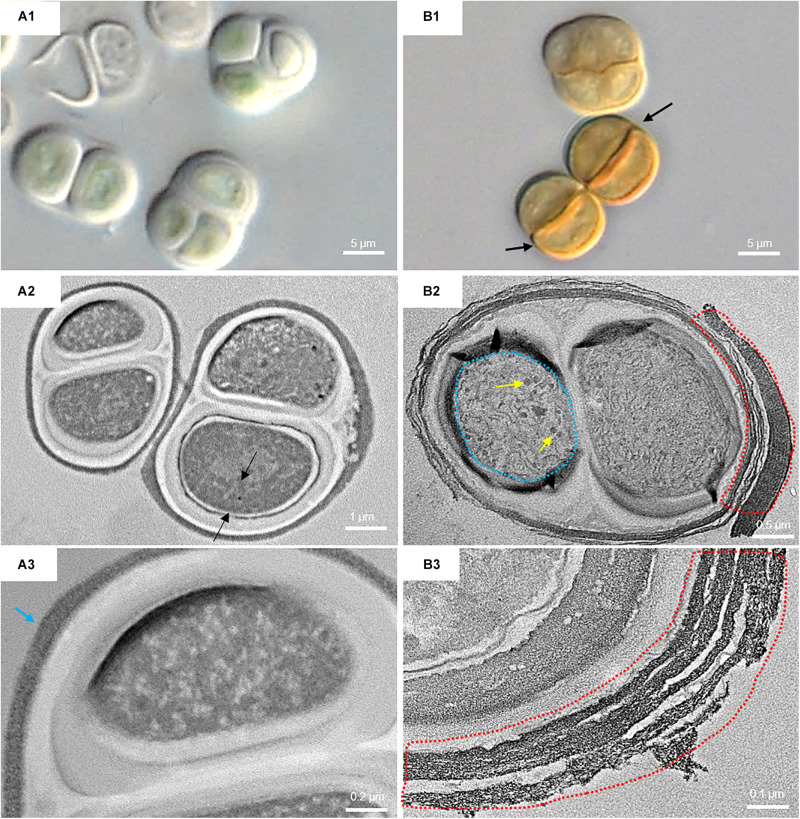
DIC microscopy and TEM images from UAM816. Series A: Cells and aggregates at the beginning of the experiment. **(A1)** DIC image of green UAM816 cells. **(A2)** TEM image showing the thylakoid arrangement (between black arrows) in PAR-only irradiated cells. **(A3)** TEM image of UAM816 cells showing a slightly more developed outermost fibrous layer (blue arrow). Series B: Cells and aggregates at their maximum scytonemin content after 9 days of irradiation with UVR + PAR. **(B1)** DIC image of brown UAM816 cells with a higher scytonemin content on the outer region. **(B2)** TEM image of cells showing disaggregation of the thylakoid membranes (blue dotted line), glycogen granules [dark spots (yellow arrows)] and a high electron-dense outermost fibrous layer (red dotted line). **(B3)** TEM image of the outer part of the cell revealing a highly fibrous outermost layer (red dotted line).

Figure 6 shows the micromorphological and ultrastructural changes in UAM813 and UAM816 cells after 9 days of exposure to UVR + PAR. The UAM813 cells changed color from light green to brownish (Figures 6A1,B1), and the thylakoids (yellow lines) became separated (Figure 6B2). The thylakoid membranes in the cells exposed to PAR only (Figure 6A2) still made intimate contact, and the cells showed a nucleoid area. The outermost layer of the EPS was more electron dense in cells after exposure to UVR + PAR (Figure 6B2) and had a granular, fibrous appearance (Figure 6B3) compared to the compact sheath observed in the UAM813 cells exposed to PAR only (Figure 6A3). When exposed to UVR + PAR for 9 days, the UAM816 cells showed evident color changes as well (Figures 7A1,B1).

In the UAM816 strain, the thylakoid membranes were beginning to disintegrate and glycogen granules were seen over them (Figures 7A2,B2). In both UAM813 and UAM816 strains an intense brownish color was observed on the outermost layer of the EPS corresponding to an electron dense matrix (Figures 6, 7A3,B3).

## Discussion

This work provides new insight into the possible behavior of endolithic cyanobacteria of the Atacama Desert. Different endolithic communities have been recorded in this desert (Wierzchos et al., 2006, 2015; Crits-Christoph et al., 2016; Meslier et al., 2018), their protective rock covering serving as the first line of defense against the damage provoked by exposure to excessive solar radiation. However, cyanobacteria may possess second and even third lines of defense (Vítek et al., 2014, 2017; Wierzchos et al., 2015), suggesting that different taxa adapted to the specific stimuli acting upon them. Certainly, while the examined endolithic *Chroococcidiopsis* strains were both isolated from endolithic habitats and areas with similar hyper-arid climatic conditions, they showed differences in their response to exposure to light.

Unlike that reported by other authors (He and Häder, 2002; Rastogi et al., 2014b; Singh et al., 2014), both strains showed strong ROS accumulation when exposed to PAR, and both responded in the longer term by producing scytonemin (Dillon et al., 2002; Fleming and Castenholz, 2007; Rajneesh et al., 2019). Both strains accumulated more ROS under PAR than under UVR + PAR (Figure 1), revealing them a fairly low acclimation to PAR in the short term. For both strains, this might justify their endolithic nature; by living in the endolithic microhabitats they would avoid such exposure. Cyanobacteria from hot springs, such as *Nostoc* and *Fischerella* (Rajneesh et al., 2019), and agricultural fields such as *Anabaena* (He and Häder, 2002) suffer no such PAR-related oxidative stress when exposed to 12 W m^–2^ PAR for 72 h and to 14.7 W m^–2^ PAR for 24 h, respectively.

The UAM813 strain was affected more strongly in terms of ROS accumulation under both PAR and UVR + PAR conditions; indeed, it continued unabated over the 3 days of exposure (Figure 1). However, in the UAM816 strain, ROS accumulation began to fall after 24 h under PAR and especially under UVR + PAR. The same is reported to occur in *Anabaena* sp. under both PAR and UVR + PAR conditions (1.07 W m^2^ UVA, 10.6 W m^2^ PAR) after 48 h (He et al., 2002) and with respect to the latter light combination (1.21 W m^–2^ UVA, 14.7 W m^–2^ PAR) but after 48 h (He and Häder, 2002), and to *Nostoc flagelliforme* after 72 h under 13 W m^–2^ PAR (Han et al., 2019). The observed stronger short-term response to PAR compared to UVR + PAR could be explained by the lack of mechanisms to cope with excess of PAR in both strains, while they would count on specific mechanisms to cope with the oxidative stress due to their exposure to UVR, as previously reported for other cyanobacteria (Rahman et al., 2014; Rastogi and Incharoensakdi, 2014).

No severe ultrastructural damage was observed in the UAM813 strain after 9 days of exposure to either PAR or UVR + PAR, although an increase in the electron dense fibrous outermost EPS layer was detected. This might be linked to the proportion of unviable and damaged cells observed after 15 days of exposure when the percentage of both types of damaged cell reached their maxima. Indeed, the high proportions reached might explain the relatively small relative content of scytonemin at 15 days compared to that seen at 9 days. The reduced number of cells able to produce scytonemin – a consequence of their reduced metabolic activity – along with a slight increase in DW due to the thickening of the EPS, might have helped maintain or slightly reduce the ratio of scytonemin in relation to DW.

The response of the UAM816 strain to long-term UVR + PAR might be explained by the changes sustained to its ultrastructure and metabolic activity. After 9 days of exposure to UVR + PAR, the ultrastructural damage sustained was greater than that inflicted upon the UAM813 cells. This timepoint coincides with the appearance of large proportions of unviable and damaged cells. The recovery of a more normal physiological status after this timepoint might be explained by the strain’s greater capacity (compared to UAM813) to eventually cope with this type of stress. This capacity to recover and acclimate to the conditions would allow an increase in the population, leading to a subsequent reduction in the relative scytonemin content; the cells in the lower layers of the population would not need to produce it since they would be protected from the light by those in the layers above.

Interestingly, both the present strains produced significantly less scytonemin than previously reported for *Chroococcidiopsis* strains from desert crusts collected in the Vizcaíno Desert (Mexico) (Dillon et al., 2002; Fleming and Castenholz, 2007).

Given the differences observed in the effects of PAR and UVR + PAR on the studied strains and their responses to these treatments, the tested variables might be able to act as indicators of light-acclimation capacity in the short and long term. A slower removal or non-removal of accumulated ROS would suggest a weaker short-term acclimation capacity. A weaker long-term acclimation capacity would be characterized by (i) longer maintenance of scytonemin production relative to DW, (ii) a reduction in the relative abundance of metabolically active cells, and (iii) ultrastructural damage.

The UAM813 cells reacted less well to both types of light treatment, which might justify its choice of cryptoendolithic microhabitat in a halite crust, which would always protect it from solar radiation. Strain UAM816, however, was isolated from a chasmoendolithic microhabitat, leaving it more exposed to direct PAR and UVR via fissures or cracks in the rock substrate. It might be expected that this strain should be better able to protect itself from this light via other mechanisms.

There could be a linkage between each strain and its original microhabitat explained by a microhabitat specific environmental pressure. Thus, *Chroococcidiopsis* strains inhabiting certain endolithic microhabitats and lithic substrates could be absent from a different endolithic microhabitat and substrate in the same desert. This differential distribution could be explained by the possession of specific adaptations and the acclimation capacity of these organisms to the specific abiotic stresses occurring in the endolithic microhabitat they are inhabiting.

The production of scytonemin by both the studied strains could have benefits for the entire endolithic communities to which they belong, with its producers providing a “parasol” to those organisms living below them. This protection would be durable given the stability of scytonemin (Dillon et al., 2002; Fleming and Castenholz, 2007; Rastogi et al., 2014a; Vítek et al., 2017) deposited within the EPS (Vítek et al., 2014) as also observed in this study (Figure 6).

The endolithic microhabitats from which the studied strains came differ slightly in terms of the amount of light that penetrates them. The effect of the light treatments on these strains, and their responses to it, suggest that each adapted in its own way according to the selection pressure it faced. Thus, *Chroococcidiopsis* strains that inhabit endolithic microhabitats in certain rock substrates might be absent from those within other rock substrates in the same desert, confirming the statement “Everything is everywhere and the environment selects” (Baas-Becking, 1934). Everything meaning, the different *Chroococcidiopsis* strains, everywhere, the endolithic microhabitats of the hyper-arid core of the Atacama Desert, and selective environment, the slight differences in direct exposure, in this case to solar radiation, between lithic substrates and the type of endolithic microhabitat.

## Data Availability Statement

The original contributions presented in the study are included in the article/Supplementary Material, further inquiries can be directed to the corresponding authors.

## Author Contributions

MC and JW designed and performed the research and conceived the original project. MC, JW, and AQ wrote the manuscript. MC, JW, and CA performed the microscopy. HM-M performed HPLC analysis. All authors contributed to editing and revising the manuscript and approved this version for submission.

## Conflict of Interest

The authors declare that the research was conducted in the absence of any commercial or financial relationships that could be construed as a potential conflict of interest.
