# Supplementary material for: Response of Endolithic Chroococcidiopsis Strains From the Polyextreme Atacama Desert to Light Radiation
Source: Front Microbiol. 2021 Jan 18;11:614875. doi: 10.3389/fmicb.2020.614875 (PMC7848079; doi:10.3389/fmicb.2020.614875)
Supplement: Supplementary file 1 [file Data_Sheet_1.PDF]

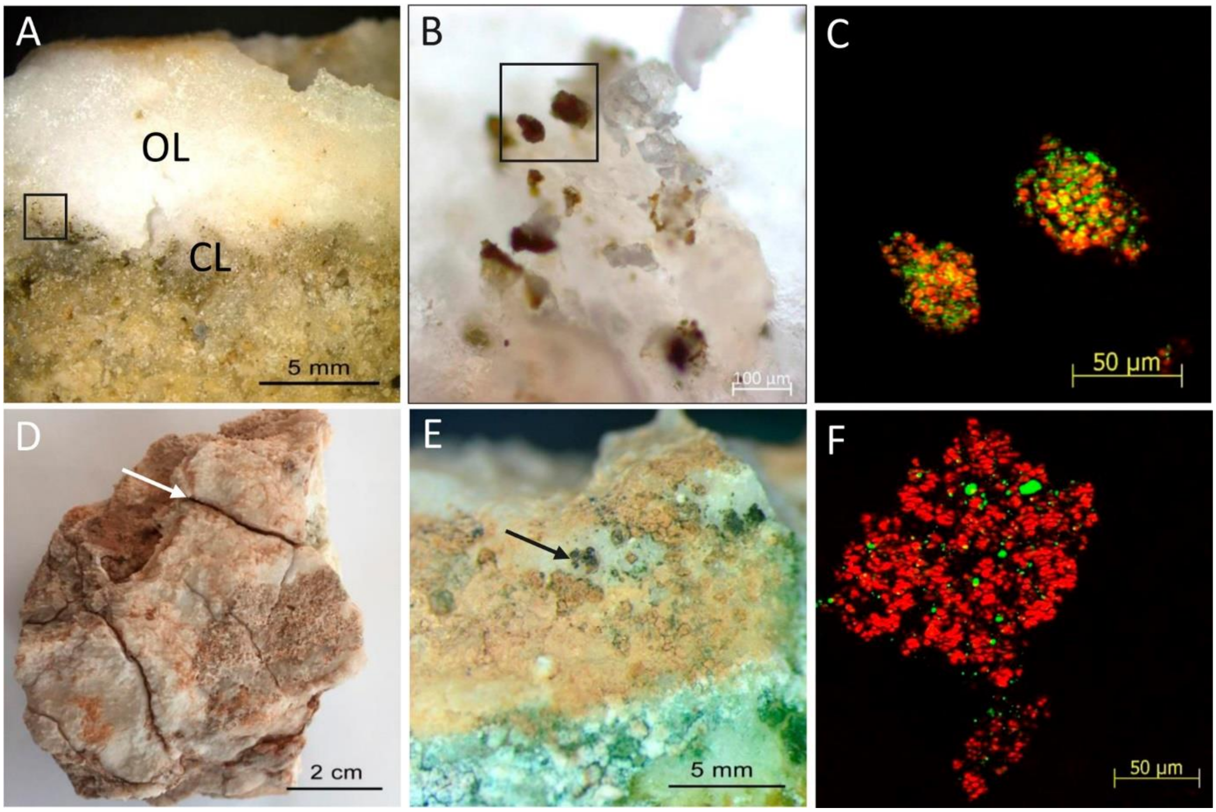

Figure S1. Stereoscopic microscopy views of sampling zones of cyanobacteria and corresponding 3D reconstruction by Structural Interference Microscopy (SIM) of cyanobacteria aggregates from two endolithic habitats. Fig. S1A show general view and Fig. 1B detailed view (square marked in Fig. S1A) of fractured halite pinnacle with a non-colonized outer layer (OL) and cryptoendolithic sampling zone (CR). Note, this dark color of cyanobacteria aggregates is due to scytonemin presence. Fig. S1C SIM image of 3D reconstruction view of two cyanobacteria aggregates (square marked in Fig. S1B). SIM was performed according to Wierzchos et al.(2011). Fig.S1D show view of fracturated surface of the calcite and arrow point to the fissure. Wall of this fissure covered by cyanobacteria is shown in Fig. S1E; arrow point to sampling area lying in upper part of the fissure and to cyanobacteria aggregate shown in 3D reconstruction image in Fig. S1F.). In both SIM images (Figs. S1C and S1F) red signal (viable cells) and green signal (degraded cells) are autofluorescence of cyanobacteria cells. Cells viability were interpreted according to Roldan et al. (2015).

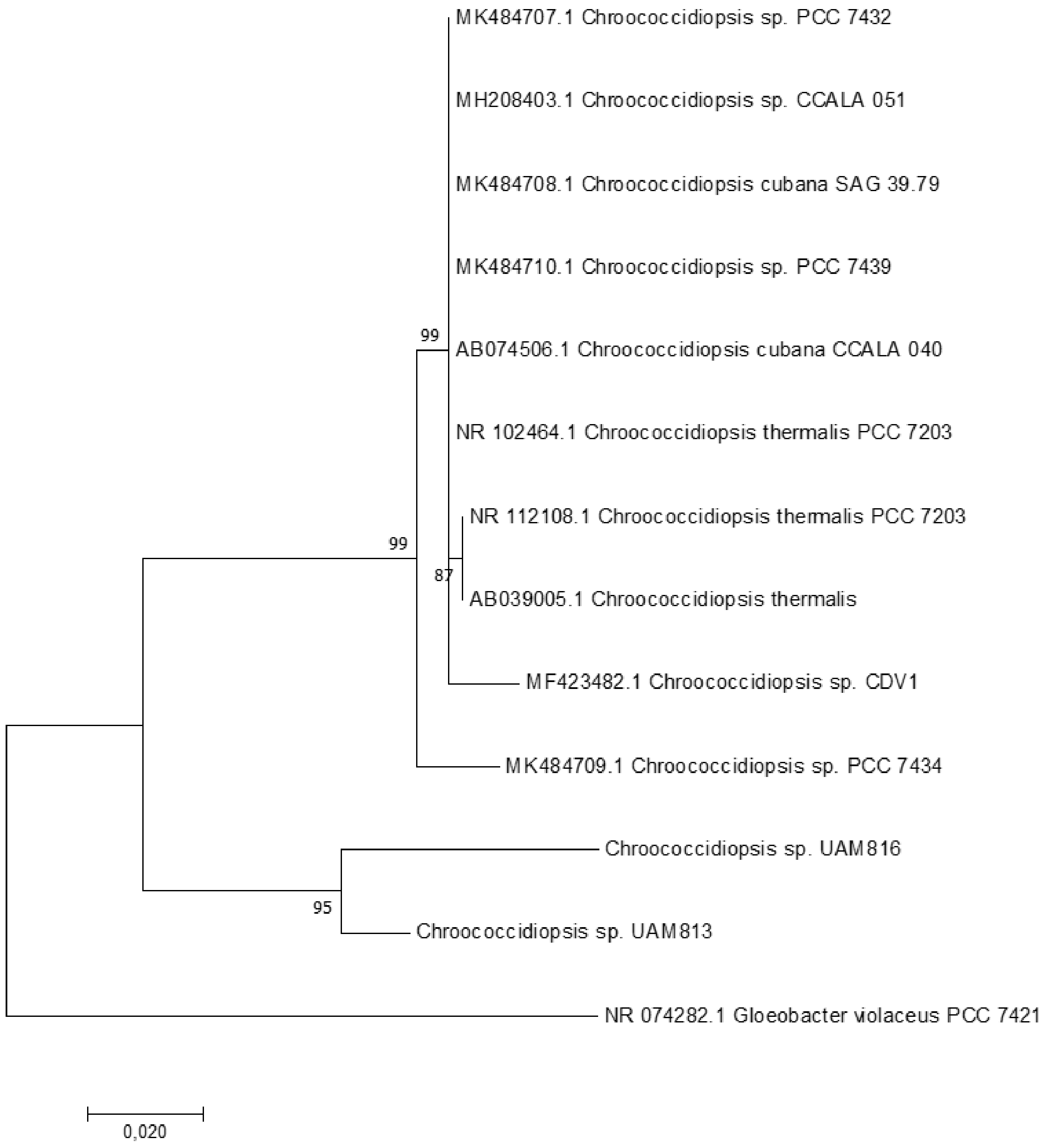

Figure S2. Maximum likelihood tree based on partial 16S rRNA sequences of closely related *Chroococcidiopsis* sequences from NCBI and from this study (UAM813, UAM816). Scale bar indicates 2% sequence divergence. Sequences from this work are available at the NCBI SRA database under the BioProject ID PRJNA637482

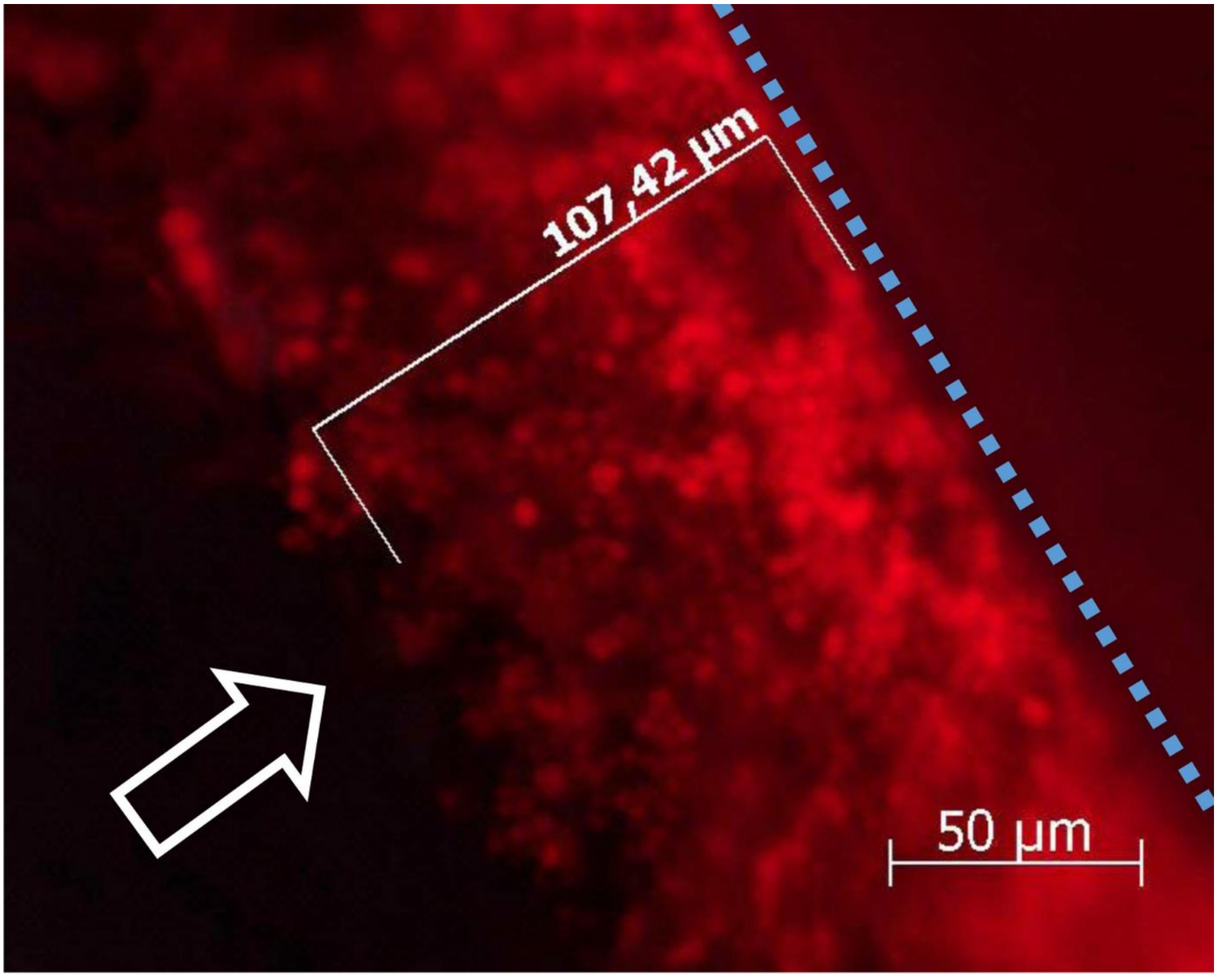

Figure S3. Cross section of *Chroococcidiopsis* UAM813 cells on filter under fluorescence microscopy. A 100µm layer of UAM813 cells on filter. Contact zone between cells and filter is indicated by a blue dotted line. Red signal shows autofluorescence of cyanobacterial cells. Empty arrow indicates the light source direction.

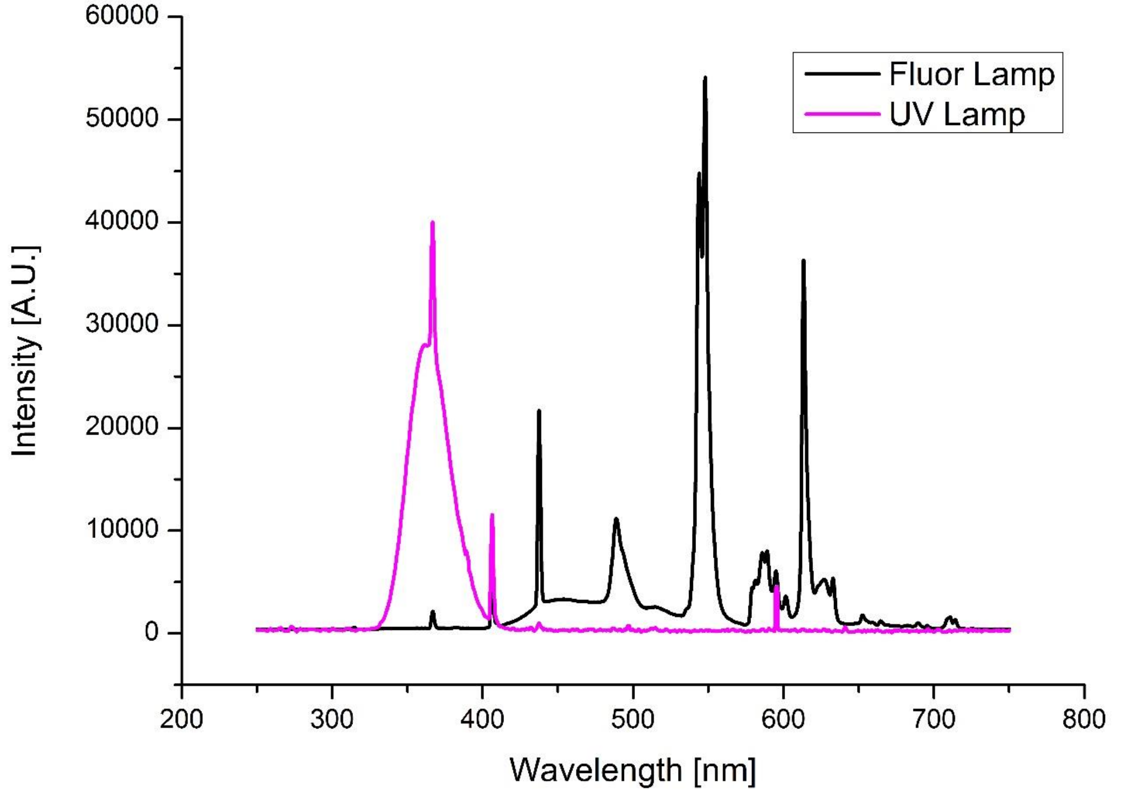

Figure S4. Lamp spectra. Fluorescent lamp (black) and UV lamp (pink).

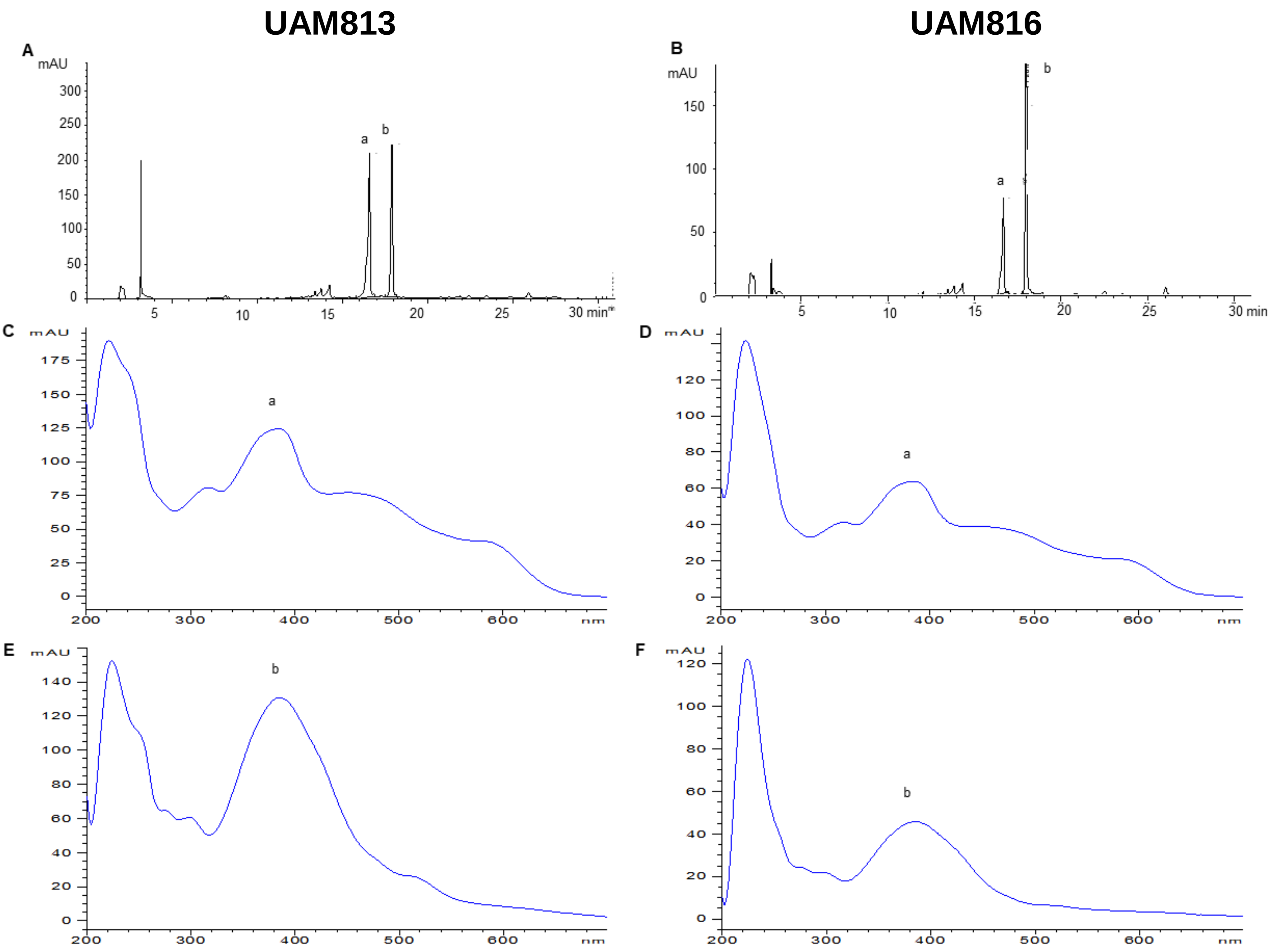

Figure S5. **The HPLC chromatogram and absorption spectra of scytonemin extract of *Chroococcidiopsis* strains UAM813** (A, C, E) **and UAM816** (B, D, F). A and B: The HPLC chromatogram of the reduced (a) and oxidized (b) scytonemin in UAM813 and UAM816. The absorption spectra of the reduced scytonemin of UAM813 (C) and UAM816 (D), and the oxidized scytonemin of UAM813 (E) and UAM816 (F)
